# Development and validation of a qPCR assay for the detection of residual host cell DNA in rabies vaccines produced in Vero cells

**DOI:** 10.3389/fbioe.2025.1611428

**Published:** 2025-10-06

**Authors:** Danhua Zhao, Weiying Zong, Wanxin Wu, Yuhua Li, Zongsong Wu, Zhixing Yang, Shouchun Cao

**Affiliations:** ^1^ NHC Key Laboratory of Research on Quality and Standardization of Biotech Products, National Institutes for Food and Drug Control, Beijing, China; ^2^ Huzhou Shenke Biotechnology Co., Ltd, Huzhou, Zhejiang, China

**Keywords:** Vero cell, rabies vaccine, exogenous DNA residue, quantitative polymerase chain reaction, method validation

## Abstract

Residual host cell DNA in biological products, including rabies vaccines, poses potential health risks such as tumorigenesis and infectivity. Regulatory authorities have set limits for residual DNA levels to ensure product safety. Among various detection methods for residual DNA, quantitative PCR (qPCR) is recognized for its high sensitivity and efficiency. This study developed and validated a qPCR assay for detecting residual Vero DNA in rabies vaccines produced in Vero cells. The assay targeted two highly repetitive Vero genomic DNA sequences: the “172bp” sequence and the Alu repetitive sequence. The method was optimized and validated for linearity, range, quantitation limit, detection limit and specificity, etc. The qPCR assay for the “172bp” sequence exhibited excellent linearity, with a quantification limit of 0.03pg/reaction and a detection limit of 0.003pg/reaction. The relative standard deviation (RSD) across samples ranged from 12.4% to 18.3%, and the recovery rate was between 87.7% and 98.5%. No cross-reactivity was observed with common bacterial and cell strains, indicating a high specificity of the assay. These findings suggest that the qPCR method is a reliable approach for quantifying residual Vero DNA in pharmaceuticals and for regulatory compliance monitoring. The assay method has been adopted by local vaccine manufacturers, and has been included in the Chinese Pharmacopoeia, thus will help to enhance vaccine quality and safety.

## 1 Introduction

Vero cell, a cell line that has been continuously passaged, is widely used for vaccine production. In 1962, two scientists, Y. Yasumura and Y. Kawakita, from Chiba University in Japan, first established the Vero cell line (Yasumura and Kawakita, 1963). The parent cells were derived from normal adult African green monkey renal epithelial cells. In 1964, Dr. B. Simizu introduced the Vero cell line at passage 93 to the Tropical Virus Research Laboratory at the NIH Institute of Allergy and Infectious Diseases (NIAID); the cell strain was cultivated to passage 113 and then submitted to the ATCC for preservation. At ATCC, the cells underwent 121 passages, and a cell bank was also established. In 2014, the complete genome of the Vero cell line was sequenced by Japanese scientists. Since the primary green monkey kidney cells contain 60 chromosomes, the number of chromosomes in the Vero cell line ranges from 52 to 62, mostly having 59 (Osada et al., 2014). Moreover, Vero is one of the cell substrates recommended by the World Health Organization (WHO) for human vaccine production. A variety of vaccine products from the Vero cell line have been successfully developed worldwide.

Biological products derived from the Vero cell line, such as Vero cell-adapted rabies vaccine (Wu et al., 2011), may contain specific impurities, including host cell DNA residues. Host cell DNA residues from biological products are associated with potential tumorigenic, infectious, and immunomodulatory risks (United States Pharmacopeia, 2025; Li et al., 2014; Li et al., 2009; André et al., 2016). Hence, WHO and US-FDA allow up to 10 ng/dose of residual DNA in biological products or vaccines (WHO, 2025; FDA, 2010). To ensure the product safety, highly sensitive and accurate methods for detecting and quantifying trace-level DNA are indispensable. Meanwhile, the established method could also help guide the downstream purification process in pharmaceutical companies and meet stringent regulatory demands.

To compare the current residual DNA detection methods, the detection limit of each method is listed in Table 1. Among these methods, quantitative polymerase chain reaction (qPCR) is a widely used fluorescence-based technique for assessing gene expression levels in real-time settings. Thus, qPCR is regarded as the most practical method for residual DNA quantification due to its high sensitivity, accuracy, precision, and time-saving characteristics, and it is also the only technique specified in Chapter 509 of the United States Pharmacopoeia (The United States Pharmacopeial Convention, 2025).

**TABLE 1:** Comparison of detection limits of different residual DNA detection methods.

Method	Detection limit (DL)
Fluorescent dye (PicoGreen)	ng (10^−9^ g) (Chinese Pharmacopoeia Commission, 2015)
Hybridization	1–10 pg (Cao et al., 2013)
Immunoenzymatic method	pg (10^−12^ g) (European Directorate for the Quality of Medicines & HealthCare, 2025)
PCR method	fg (10^−15^ g) (European Directorate for the Quality of Medicines and HealthCare, 2025)

The aim of this study is to develop a qPCR-based method for detecting residual Vero cell DNA. This method will be a powerful tool for users to guide the downstream purification process and provide quality control during manufacturing.

## 2 Materials and methods

### 2.1 Cell culture

The Vero cell line was purchased from Cell Bank/Stem Cell Bank, Chinese Academy of Sciences. Cells were cultured in Eagle’s minimum essential medium (Gibco, Waltham, MA) supplemented with 10% fetal bovine serum (FBS, Gibco) and 5% CO_2_ at 37 °C and passaged every 2–3 days. Other cell lines (CHO, HEK293T, HEK293, NS0, and MDCK) and common bacterial strains (*E.coli* and *Pichia pastoris*) were provided by Huzhou Shenke Biotechnology Co., Ltd. (HZSKBio, China).

### 2.2 Reagents

The DNA preparation kit (magnetic beads method) (HZSKBio, China) and in-house detection reagents (containing enzymes, buffers, probes, and primers) were purchased from HZSKBio. Vaccine drug substance (DS) samples, Vero DNA standard, and Vero DNA National Standard for the hybridization method were provided by the National Institutes for Food and Drug Control (NIFDC). For the specificity test, genomic DNA was extracted from fresh cell cultures using the Genomic DNA Buffer Set and Genomic-tip 500/G (QIAGEN, Germany).

### 2.3 Bioinformatic analysis

The key features of qPCR highlighted suitable target sequences, primer and probe design, and an optimized reaction system. The specific target sequence should meet the following requirements: 1. the sequence is unique to the genome of the Vero cell line; 2. the copy number is closely correlated to the fragment size of the target sequence within the Vero cell line genome; and 3. the sequence is less affected by inactivating agents, such as β-propiolactone and formaldehyde.

The haploid genome of primary green monkey kidney cells is 6.1 × 10^9^ bp. After analyzing the characteristics of the African green monkey genome, two highly repetitive sequences of Vero genomic DNA were selected as the amplification targets.

a. “172 bp” sequence (GenBank: V00145.1). Based on the literature, the genome of the Vero cell line contains a unique 172 bp tandem repeat sequence, which is counted as 6.8 × 10^6^ copies/haploid genome (Singer, 1979). Primers were designed to detect fragments of 99 bp and 154 bp on the “172 bp” sequence.

The primers and probe information for the 99 bp amplicon was as follows:

Forward primer: 5′-CTG​CTC​TGT​GTT​CTG​TTA​ATT​CAT​CTC-3′

Reverse primer: 5′-AAA​TAT​CCC​TTT​GCC​AAT​TCC​A-3′

Probe: 5′-CCTTCAAGAAGCCTTTCGCTAAG-3′

The primers and probe information for the 154 bp amplicon was as follows:

Forward primer: 5′-GCT​TTC​TGA​GAA​ACT​GCT​CTG​TGT-3′

Reverse primer: 5′-GGA​AGA​TAT​TTC​CTT​TTT​CAC​CAT​AGC-3′

Probe: 5′-CCTTCAAGAAGCCTTTCGCTAAG-3′

b. Alu repetitive sequence (GenBank: X01476.1). Based on the literature (Grimaldi et al., 1981), the Alu repetitive sequence (293 bp) of the Vero cell line genome is approximately 3 × 10^5^ copies/haploid genome. Primers were designed to detect the fragments of 151 bp and 221 bp on the Alu repetitive sequence.

The primers and probe information for the 151 bp amplicon was as follows:

Forward primer: 5′-ACG​GTG​AAA​CCC​CGT​CTC​TA-3′

Reverse primer: 5′-GCG​GTG​GCC​GGA​TCT​C-3′

Probe: 5′-CTAGCCGGGCGAGGTGGCAG-3′

The primers and probe information for the 221 bp amplicon was as follows:

Forward primer: 5′-AAT​CCC​AGC​ACT​TTG​GGA​GG-3′

Reverse primer: 5′-GCG​GTG​GCC​GGA​TCT​C-3′

Probe: 5′-CTAGCCGGGCGAGGTGGCAG-3′

### 2.4 Assay design

As described above, amplicons of 99 bp and 154 bp for the “172 bp” sequence and 151 bp and 221 bp for the Alu repetitive sequence were selected. Parameters for the standard curve were assessed, which contained 0.3 fg/μL, 3 fg/μL, 30 fg/μL, 300 fg/μL, 3 pg/μL, and 30 pg/μL for the “172 bp” sequence and 3 fg/μL, 30 fg/μL, 300 fg/μL, 3 pg/μL, 30 pg/μL, and 300 pg/μL for the Alu repetitive sequence. Residual DNA from samples was extracted using the DNA preparation kit (magnetic beads method, HZSKBio) according to the manufacturer’s instructions.

A total reaction volume of 30 μL is prepared as follows: 17 μL of qPCR buffer (containing enzymes, dNTPs, probes, and primers), 1 μL each of the forward and reverse primers, 1 μL of probe, and 10 μL of DNA standard. The reaction program was as follows: 95 °C for 10 min, followed by 40 cycles of 95 °C for 15 s and 60 °C for 1 min. qPCR validation tests were performed using the SHENTEK-96S (HZSKBio) instrument, unless mentioned specifically.

### 2.5 Method validation

#### 2.5.1 Linearity and range

The standard curve was prepared from a 10-fold dilution series of Vero genomic DNA. Then, the linearity (*R* ^2^ value), amplification efficiencies for the standard curves, relative standard deviation (RSD), and relative bias of samples were assessed.

#### 2.5.2 Quantitation limit

For the quantitation limit (QL), the standard curve was generated using 10-fold serial dilutions of standard DNA from 30 pg/μL to 0.003 pg/μL in TE buffer. Each standard was analyzed in triplicate. The selected Vero DNA standard concentration was tested in 10 replicates for the QL test.

#### 2.5.3 Detection limit

Vero DNA standard was diluted in TE buffer to an appropriate concentration for the detection limit (DL) test and tested in eight replicates. Three independent experiments were performed, and the detection limit was determined accordingly.

#### 2.5.4 Specificity

To assess the specificity of the qPCR assay, genomic DNA from various species was analyzed. Consequently, DNA samples from seven different cell lines, namely, CHO, *E. coli*, HEK293, HEK293T, Pichia pastoris, NS0, and MDCK, were extracted and adjusted to a uniform concentration of 300 pg/μL for the specificity test.

#### 2.5.5 Accuracy

Samples spiked with different concentrations of Vero genomic DNA (30 pg/μL, 0.3 pg/μL, and 0.006 pg/μL) were extracted using the DNA preparation kit (HZSKBio) and tested, and spike recovery was assessed in parallel.

#### 2.5.6 Repeatability

Samples spiked with different concentrations of Vero genomic DNA (3 pg/μL and 0.03 pg/μL) were extracted using the DNA preparation kit (HZSKBio) and tested for six replicates sequentially, and RSD was assessed.

#### 2.5.7 Intermediate precision

Samples with 30 pg/μL, 0.3 pg/μL, and 0.006 pg/μL of Vero genomic DNA were extracted and tested by three technicians for total three individual results, and RSD was assessed.

#### 2.5.8 Robustness

Quantification limit was assessed using qPCR systems from different providers, including SHENTEK-96S (HZSKBio), ABI7500 (Thermo, Waltham, MA), LightCycler 480 II (Roche, Basel, Switzerland), CFX96 (Bio-Rad, Hercules, CA), FQD-96A (Bioer, Hangzhou, China), and qTOWER 3G (Jena, Jena, Germany).

Vero genomic DNA was sonicated for a different duration to obtain a series of DNA fragments (Sonicator, KQ-800DE, power 320W). DNA fragment size was assessed through electrophoresis, and the influence of the DNA length was analyzed using qPCR.

### 2.6 Data analysis

#### 2.6.1 Calculation of the amplification efficiency

$$E=\left(10^{-1/\text{slope}}-1\right)\times 100\%.$$

The slope is derived from the standard curve by plotting Ct values against the logarithm of the initial template quantity.

#### 2.6.2 Calculation of sample RSD

RSD=Standard deviation of detection value / mean detection value×100%.

#### 2.6.3 Calculation of the residual concentration of sample DNA

$$\text{Residual DNA concentration of sample}=\frac{\text{mean}\;\text{detection}\;\text{value}\times \text{elution}\;\text{volume}}{\text{sample}\;\text{volume}}.$$

#### 2.6.4 Relative bias calculation

$$\text{Relative bias }\left(\%\right)=\left(\text{Detection value}-\text{Theoretical value}\right)\;/\;\text{Theoretical}\text{ value}\times 100\%.$$

#### 2.6.5 Recovery calculation

$$\text{Recovery}=\left(\text{Mean}\;\text{observed}\;\text{DNA}\;\text{of}\;\text{spiked}\;\text{sample}-\;\text{Mean}\;\text{observed}\;\text{DNA}\;\text{of}\;\text{unspiked}\;\text{sample}\right)/\text{Expected}\;\text{DNA}\;\times 100\%.$$

## 3 Results

### 3.1 Assay optimization

Vero genomic DNA showed superior linearity for all four amplification targets. The “172 bp” sequence yielded higher DNA concentrations for all three rabies vaccine DS solutions because it contained 10 times more repeat counts than the Alu repetitive sequence in the Vero genome. The detection value is stable for both the target sequences using the Vero DNA National Standard for the hybridization method, suggesting that the established detection method is accurate and reliable. In 2010, FDA suggested that residual DNA fragments should be smaller than approximately 200 base pairs, which is less than the length of a functional gene (FDA, 2010). Given the fact that the potential increased risk is associated with residual DNA fragments larger than 200 bp, the 154 bp fragment from the “172 bp” sequence was selected for further validation as it is closer to the 200 bp threshold.

#### 3.1.1 qPCR method for the “172 bp” sequence target gene

**FIGURE 1:**
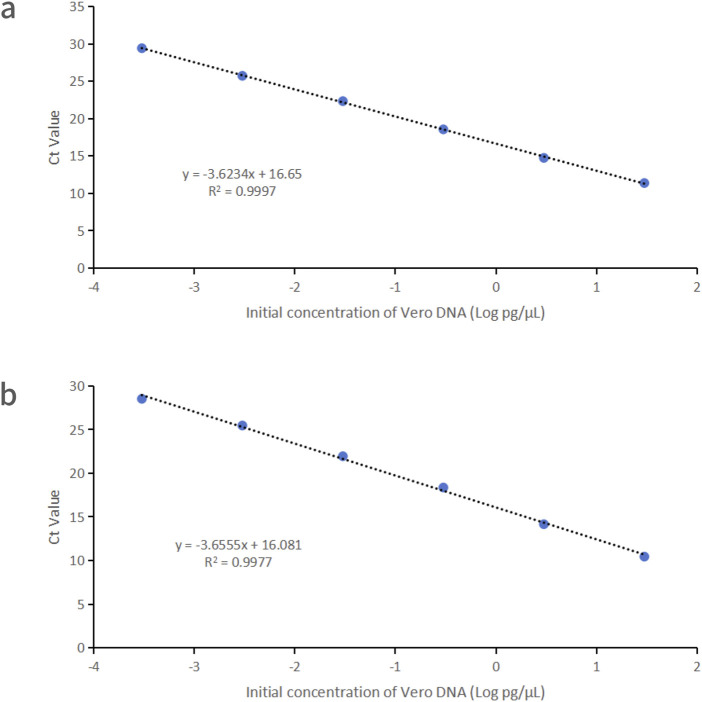
Linearity graphs for Vero DNA standards based on the average cycle of Ct values plotted against the logarithm of the standard concentration corresponding to two distinct amplicon sizes. Each plot represents the average of triplicate Ct values (n = 3). **(a)** 99 bp amplicon size. **(b)** 154 bp amplicon size. Linearity was determined using qPCR with specific primers and probes designed for each amplicon.

Vero DNA demonstrated excellent linearity across six orders of magnitude (0.3 fg/μL–30 pg/μL), with correlation coefficients of 0.9997 and 0.9977 for the 99 bp and 154 bp amplicons, respectively. Corresponding amplification efficiencies were 88.8% and 87.7%, respectively (Figure 1).

#### 3.1.2 qPCR method for the Alu-like sequence target gene

**FIGURE 2:**
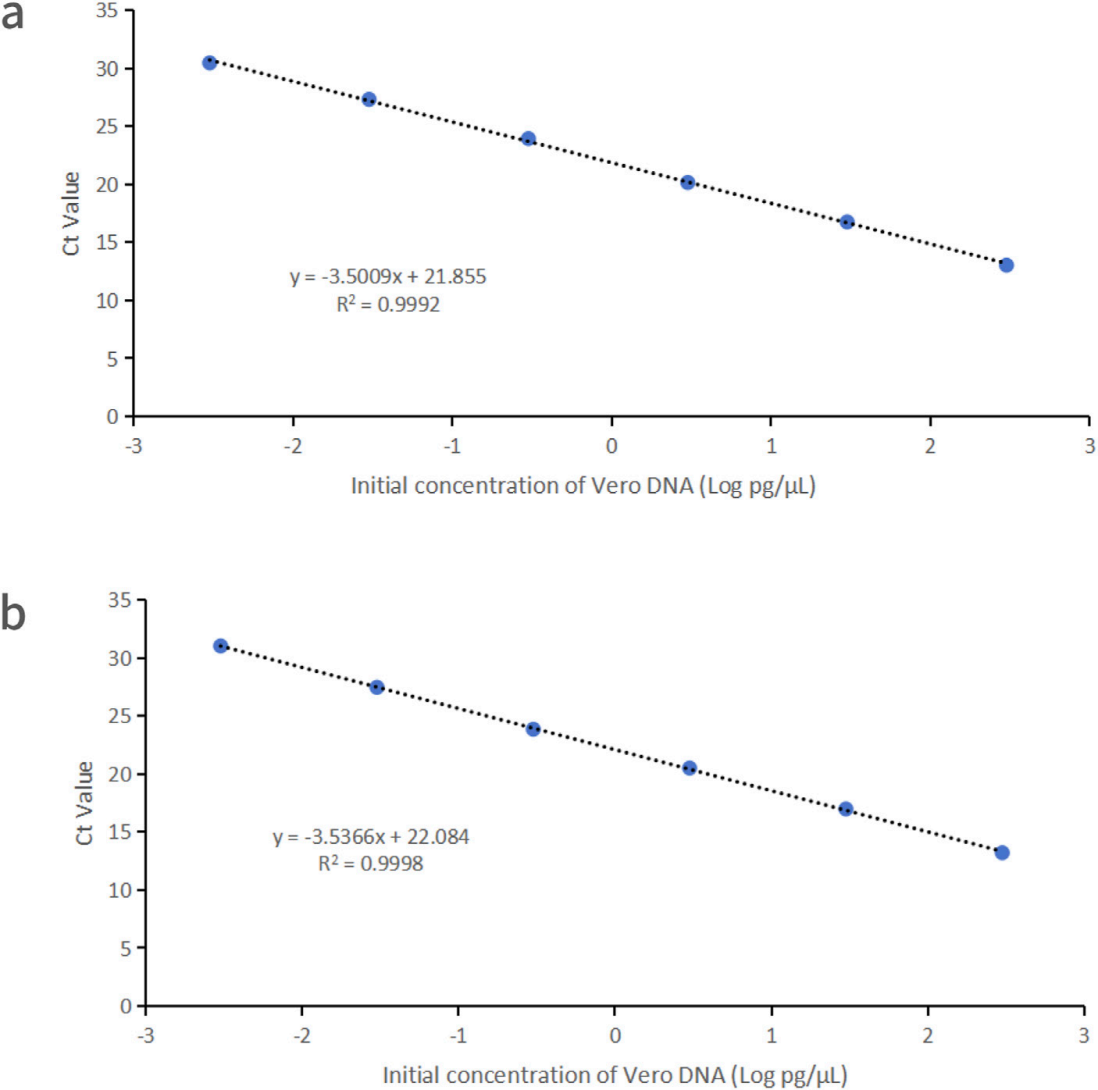
Linearity graphs for Vero DNA standards based on the average cycle of Ct values plotted against the logarithm of the standard concentration corresponding to two distinct amplicon sizes. Each plot represents the average of triplicate Ct values (n = 3). **(a)** 151 bp amplicon size. **(b)** 221 bp amplicon size. Linearity was determined using qPCR with specific primers and probes designed for each amplicon.

Vero DNA demonstrated excellent linearity across six orders of magnitude (3 fg/μL–300 pg/μL), with correlation coefficients of 0.9992 and 0.9998 for the 151 bp and 221 bp amplicons, respectively. Corresponding amplification efficiencies were 93.0% and 91.8%, respectively (Figure 2).

#### 3.1.3 Detection of the vaccine drug substance sample

**FIGURE 3:**
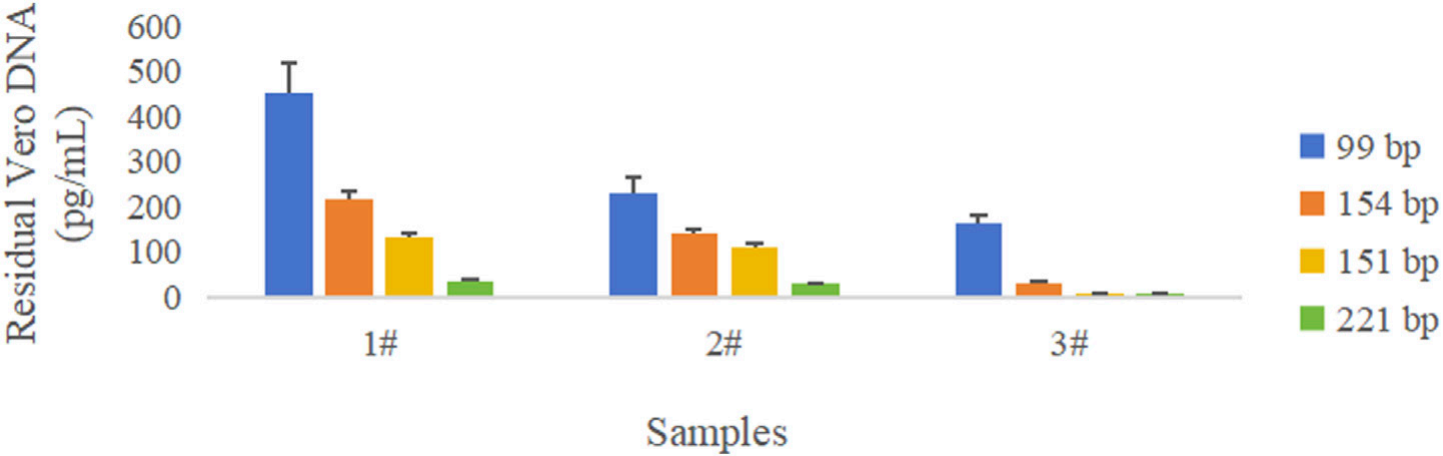
Comparison of quantity of DNA amplified with different amplicon lengths in three rabies vaccine drug substances. Blue color represents the 99 bp amplicon from the “172 bp” sequence, orange color represents the 154 bp amplicon from the “172 bp” sequence, yellow color represents the 151 bp amplicon from the Alu repetitive sequence, and green color represents the 221 bp amplicon from the Alu repetitive sequence. The error bar represents the standard deviation (SD) of the mean value.

Three rabies vaccine DS solutions (Sample 1#, Sample 2#, and Sample 3#) from three vaccine manufacturers and one Vero DNA National Standard for the hybridization method were extracted using the DNA preparation kit (HZSKBIO) and tested using four amplification targets; the results are shown in Tables 2, 3 and Figure 3.

**TABLE 2:** Test results of rabies vaccine DS solution. Three samples were detected in triplicate, and the data were averaged using all four detection reactions. The recovery rate was calculated according to the formula in Section 2.6.

Sample	Residual concentration (pg/mL)	Recovery (%)	Amplicon size, bp	Target sequence
1#	451	86	99	“172 bp” sequence
	215	69.5	154	
	130	121.3	151	Alu repetitive sequence
	33.5	83.2	221	
2#	228	86.5	99	“172 bp” sequence
	140	83.1	154	
	109	81.1	151	Alu repetitive sequence
	27.7	75.8	221	
3#	161	91	99	“172 bp” sequence
	31.5	72.3	154	
	8.39	101.7	151	Alu repetitive sequence
	0.263	74	221	

**TABLE 3:** Test results of the Vero DNA National Standard for the hybridization method. The sample was detected in triplicate, and the DNA concentration was averaged using four amplification targets. The recovery rate and RSD were calculated according to the formula in Section 2.6.

Sample	Recovery (%)	DNA concentration (pg/mL)	RSD (%)	Amplicon size, bp	Target sequence
Vero DNA National Standard for hybridization method	77.3	6.80 × 10^4^	9.1	99	“172 bp” sequence
	70.7	5.58 × 10^4^		154	
	75.4	6.49 × 10^4^		151	Alu repetitive sequence
	73.3	6.82 × 10^4^		221	

The RSD for the DNA concentration across the four groups was 9.1%, indicating that testing of the Vero DNA National Standard using the hybridization method was not affected by the amplicon size. This may be related to the intact nature of the Vero genomic DNA.

The results showed that the copy number of short amplicons was high, and longer amplicons were fewer in the same sample; the larger the amplified fragment, the higher possibility of leaked detection could occur.

RSD for the DNA concentration across four groups was 9.1%, indicating that the testing of the Vero DNA National Standard for hybridization method was not affected by the amplicon size. This may be related to the intact nature of the Vero genomic DNA.

### 3.2 Method validation results

Based on the results above, subsequent method validation was performed with the 154 bp amplicon from the “172 bp” sequence.

#### 3.2.1 Linearity and range

**FIGURE 4:**
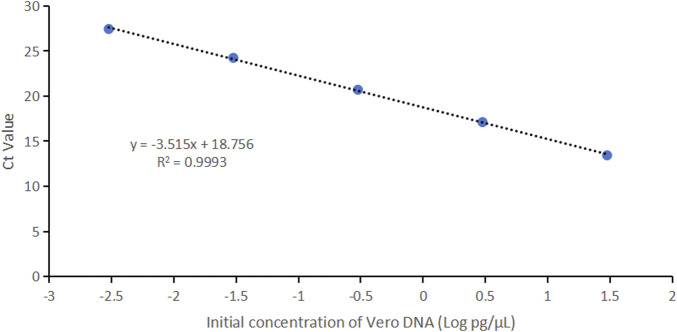
Linearity graph for Vero DNA standards based on the average cycle of Ct values plotted against the logarithm of the standard concentration. Each plot represents the average of triplicate Ct values (n = 3). The linearity was determined using qPCR with specific primers and probe designed for the 154 bp amplicon.

Vero genomic DNA showed superior linearity across concentrations of 3 fg/μL, 30 fg/μL, 300 fg/μL, 3 pg/μL, and 30 pg/μL, with a correlation coefficient of 0.9993, an amplification efficiency of 92.5%, and high precision and accuracy (Figure 4; Table 4).

**TABLE 4:** Results for linearity and range tests. Six samples were detected in triplicate, and the mean value is shown in the table. RSD and relative bias were calculated according to the formula in Section 2.6.

Sample	Theoretical concentration (pg/μL)	Mean value (pg/μL)	RSD (%)	Relative bias (%)
1	3.00 × 10^1^	3.09 × 10^1^	4.7	3.1
2	3.00	2.73	0.4	−9.0
3	3.00 × 10^−1^	2.64 × 10^−1^	0.9	−11.8
4	3.00 × 10^−2^	2.75 × 10^−2^	2.5	−8.5
5	3.00 × 10^−3^	3.03 × 10^−3^	10.2	0.9
6	3.00 × 10^−4^	3.12 × 10^−4^	87.0	4.0

#### 3.2.2 Quantitation limit

The standard curve was generated with Vero genomic DNA (30 pg/μL−0.003 pg/μL). Diluted DNA standard of 3.00 × 10^-3^pg/μL was tested 10 times. Data were pooled to calculate QL. As shown in Table 5, the QL of qPCR assay was 3.00 × 10^-3^pg/μL (3.00 × 10^-2^pg/reaction) with a RSD value of 9.0% and relative bias of −1.6%.

**TABLE 5:** Results for the QL test. The sample was tested in 10 replicates, and the mean value is shown in the table.

Test item	Value
Theoretical concentration (pg/μL)	3.00 × 10^−3^
Test concentration (pg/μL)	2.93 × 10^−3^
	2.78 × 10^−3^
	2.88 × 10^−3^
	2.98 × 10^−3^
	3.05 × 10^−3^
	3.11 × 10^−3^
	2.62 × 10^−3^
	2.54 × 10^−3^
	3.33 × 10^−3^
	3.32 × 10^−3^
Average concentration (pg/μL)	2.95 × 10^−3^
RSD (%)	9.0
Relative bias (%)	−1.6

#### 3.2.3 Detection limit

**FIGURE 5:**
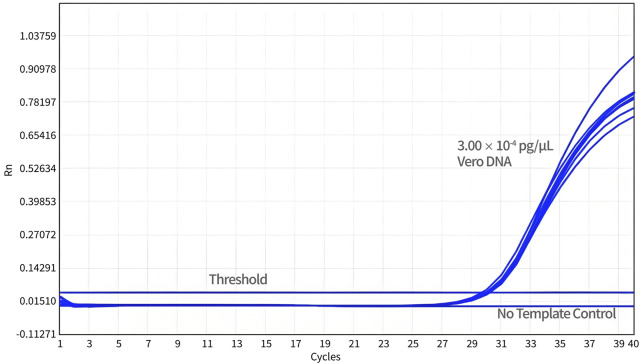
Determination of the detection limit. Diluted Vero DNA standard (DL) was assayed using qPCR with specific primers and probe designed for 154 bp primers and probe sets. The no-template control (NTC) shows no amplification peak, while all DL samples represent normal amplification curves.

For the DL test, a total of 24 datasets were obtained from three independent experiments. As shown in Figure 5, the DL of qPCR assay was 3.00 × 10^-3^ pg/reaction with a detection rate of 100%.

#### 3.2.4 Specificity

The specificity test was performed to detect any interference in Vero DNA detection by genomic DNA (3 ng/reaction) from CHO, E. coli, HEK293T, HEK293, *Pichia pastoris*, NS0, and MDCK. As shown in Table 6, the detection value of the interference DNA was lower than the quantitation limit, suggesting good specificity for the assay.

**TABLE 6:** Result for the specificity test. Seven samples were detected in triplicate, and the mean value is shown in the table.

Interfering DNA	Mean value (pg/μL)
CHO	2.32 × 10^−4^
*E.coli*	<1.000 × 10^−5^
HEK293T	<1.000 × 10^−5^
HEK293	<1.000 × 10^−5^
*Pichia pastoris*	2.57 × 10^−5^
NS0	<1.000 × 10^−5^
MDCK	1.15 × 10^−5^

#### 3.2.5 Accuracy

Assay accuracy was assessed by calculating the spike recovery rate. In this part, spiked Vero genomic DNA was spiked into TE buffer at final concentrations of 300, 3, and 0.06 pg/μL, then extracted, and assayed using qPCR in triplicate. The recovery values ranged from 87.7% to 98.5% with RSD <25% (Table 7).

**TABLE 7:** Results for the accuracy test. Three samples were extracted and detected in triplicate, and the mean value is shown in the table. RSD and spike recovery were calculated according to the formula in Section 2.6.

Expected DNA (pg/μL)	Observed DNA, mean (pg/μL)	Observed DNA, RSD (%)	Spike recovery (%)
3.00 × 10^1^	2.94 × 10^1^	11.0	98.0
3.00 × 10^−1^	2.63 × 10^−1^	3.6	87.7
6.00 × 10^−3^	6.05 × 10^−3^	9.3	98.5

#### 3.2.6 Precision

Repeatability was assessed using two samples, each with six replicates in a single experiment that included both DNA extraction and qPCR analysis. As shown in Table 8, the RSD values for these samples ranged from 6.2% to 7.0%. On the other hand, intermediate precision was evaluated using three samples, each analyzed by two analysts across three independent runs over 3 days, producing a total of nine replicates per sample. As shown in Table 9, the RSD for these samples ranged from 12.4% to 18.3%.

**TABLE 8:** Results for the repeatability test. Two samples were extracted and detected in six replicates, and the mean value is shown in the table. RSD was calculated according to the formula in Section 2.6.

Theoretical value (pg/μL)	Mean value (pg/μL)	RSD (%)
3.00	2.82	7.0
3.00 × 10^−2^	2.54 × 10^−2^	6.2

**TABLE 9:** Results for the intermediate precision test. Three samples were extracted and analyzed in nine replicates across three independent runs, for a total of nine measurements, and the mean value is shown in the table. RSD was calculated according to the formula in Section 2.6.

Theoretical value (pg/μL)	Mean value (pg/μL) (n = 9)	RSD (%)
3.00 × 10^1^	2.50 × 10^1^	16.7
3.00 × 10^−1^	2.35 × 10^−1^	12.4
6.00 × 10^−3^	5.13 × 10^−3^	18.3

#### 3.2.7 Robustness

The quantification limit was evaluated across six instruments from different suppliers, and each sample was tested for 10 replicates. The results indicated that the QL could reach 3.00 × 10^-3^pg/μL (3.00 × 10^-2^pg/reaction) on all six instruments, namely, SHENTEK-96S, CFX96, ABI7500, FQD-96A, LightCycler 480 II, and qTOWER-3G (Table 10).

**TABLE 10:** Results for the robustness test. The QL sample was detected in 10 replicates across six instruments. RSD and relative bias were calculated according to the formula in Section 2.6.

Instrument model	Theoretical value (pg/μL)	RSD (%)	Relative bias (%)
SHENTEK-96S	3.00 × 10^−3^	9.0	−1.6
ABI 7500	3.00 × 10^−3^	17.9	−6.8
CFX-96	3.00 × 10^−3^	15.4	10.8
FQD-96A	3.00 × 10^−3^	16.7	7.0
LightCycler 480 II	3.00 × 10^−3^	17.3	−13.0
qTOWER-3G	3.00 × 10^−3^	12.2	5.7

**FIGURE 6:**
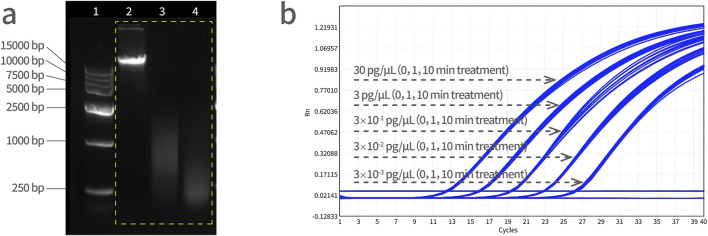
Vero genomic DNA fragmentation analysis. **(a)** Electrophoresis of DNA fragments with increasing sonication treatment time. Lane 1 is a DNA marker, lane 2 is a control sample (0 min), lane 3 is a 1 min-treated ultrasonic sample, and lane 4 is a 10 min-treated ultrasonic sample. DNA molecular weight standard: 15,000 bp, 10,000 bp, 7,500 bp, 5,000 bp, 2,500 bp, 1,000 bp, and 250 bp from top to bottom. **(b)** The Vero DNA samples were subjected to ultrasonication for 0, 1, and 10 min, followed by qPCR amplification simultaneously, and the amplification curves were analyzed together with the concentration gradient of 30 pg/μL, 3 pg/μL, 3 × 10^−1^ pg/μL, 3 × 10^−2^ pg/μL, and 3 × 10^−3^ pg/μL.

Three tubes of Vero DNA (50 μL, 30 ng/μL) were prepared by ultrasonication. To obtain a series of DNA size fragments, one tube served as a control (0 min), and the rest of two tubes were subjected to ultrasonic fragmentation for 1 min and 10 min, respectively. Standard curves were prepared from a 10-fold dilution series of these Vero DNA (sonicated for 0 min, 1 min and 10 min, respectively) and assayed by qPCR. As shown in Figure 6, the amplification curves from three fragmented DNA samples in five dilutions, all treatment groups were overlapped at each dilution, indicating that there was no significant difference on qPCR amplification with fragmented DNA and the PCR reaction system is not affected by the size of DNA fragments.

## 4 Discussion

In recent years, the qPCR method has been widely used in the routine detection of residual host cell DNA content determination due to its high specificity, high sensitivity, wide linear range, and rapid and high-throughput characteristics (Peper et al., 2014; Varnamkhasti et al., 2021; Zheng et al., 2019).

In China, dot blot hybridization was once the conventional method for detecting host residual DNA (Li et al., 2008). This method is semi-quantitative and involves relatively complex operational steps. Therefore, this study explores the application of qPCR for the purpose.

For residual Vero cell DNA quantification, we established qPCR methods for the “172 bp” sequence and the Alu repetitive sequence as target genes. The assay showed typical amplification curves, with good specificity and sensitivity.

The 154 bp detection reaction from the “172 bp” sequence was chosen for Vero DNA detection. The following points are considered: first, the “172 bp” sequence had 10 times more copies in the Vero genome than the Alu repetitive sequence; thus, a higher amount of residual DNA may be detected to achieve better control of risks such as carcinogenicity and infectivity caused by residual DNA. Second, the “172 bp” sequence in Vero cells predominantly manifests as long tandem repeats, along with unrelated sequences. A maximum of 29 long tandem repeats are observed, while the unrelated sequences account for approximately 37% of the entire genome (Singer, 1979). This distinct fragment is unique to the Vero cell genome, and the distribution of this sequence, along with its derivatives, is considered a comprehensive representation of Vero cells. Thus, the “172 bp” sequence can serve as a reliable marker for the quantitative assessment of residual Vero DNA (Funakoshi et al., 2017). Third, the FDA recommends that residual DNA fragments should not exceed 200 base pairs (bp) in length. Potential residual DNA fragments containing full-length 154 bp targets of the “172 bp” sequence can be detected using the 154 bp assay, providing scientific validity for safety considerations. Although both the 154 bp and 99 bp assays offer adequate sensitivity and range, the 99 bp assay values were 1.63–5.11 times higher than those of the 154 bp assay when evaluating actual DS samples. Thus, since the 154 bp assay already meets the FDA requirement that host cell DNA fragments should not exceed 200 bp and to avoid imposing excessive demands on manufacturing enterprises, which could lead to significant resource investments and potentially impact vaccine supply, we selected the 154 bp assay targeting the “172 bp” sequence for further study.

The assay targeting a 154 bp fragment from the “172 bp” sequence demonstrated good performance in terms of linearity, range, accuracy, precision, quantitation limit, specificity, and robustness. Therefore, it is worth being adopted for monitoring residual Vero DNA in pharmaceutical enterprises and regulatory authorities.

As a well-established experimental technique, qPCR can yield varying results depending on the selection of primers and probes, including differences in the length of the amplified fragments. Few publications mentioned similar qPCR primer and probe sets for residual Vero DNA detection or other host cells; the sensitivity and range are also comparable to the current study (Vernay et al., 2019; André et al., 2016; Zhang et al., 2014). However, the efficiency of each qPCR assay is difficult to compare unless a head-to-head comparison is launched. The choice of different primers and probes offers distinct advantages and specific scopes of application. If the methods are validated and deemed suitable for domestic vaccine production processes, they can be effectively utilized.

In China, there are more than 10 manufacturers producing rabies vaccines for human use, each using distinct production processes, particularly regarding excipients. After extensive validation and exploratory studies, the method established in this research has proven suitable for quality control of domestic vaccines. It was published in the 2020 edition of the Chinese Pharmacopoeia (Chinese Pharmacopoeia Commission, 2020) and has also been commercialized as a kit for use by rabies vaccine manufacturers to enhance vaccine quality. Additionally, the 154 bp reaction was successfully used to control residual host cell DNA during continuous production of Vero cell rabies virus vaccine (Li et al., 2024).

After 5 years of practical application, the method was widely adopted by Chinese vaccine manufacturers and played a crucial role in ensuring the monitoring of residual Vero cell DNA in rabies vaccines. With its excellent detection performance and reliability, the method not only accumulated extensive application cases but also provided a strong guarantee for the continuous improvement of vaccine quality.

The method remains included in Chapter 3407 of the 2025 edition of the Chinese Pharmacopoeia, which will be implemented starting from 1 October 2025. It continues to safeguard the quality control of rabies vaccines, ensuring that every dose of vaccine meets stringent quality standards and thereby fortifying the safety for public health.

## Data Availability

The original contributions presented in the study are included in the article/supplementary material; further inquiries can be directed to the corresponding authors.
